# The prevalence and associated factors of *Helicobacter pylori* infection among asymptomatic adolescent schoolchildren in Sudan: a cross-sectional study

**DOI:** 10.1186/s12887-023-04411-5

**Published:** 2023-11-21

**Authors:** Shimos A. Alshareef, Ahmed A. Hassan, Dina N. Abdelrahman, Ashwaq AlEed, Abdullah Al-Nafeesah, Ishag Adam

**Affiliations:** 1 Virology Department, Central Laboratory, Ministry of Higher Education, Khartoum, Sudan; 2 Faculty of Medicine, University of Khartoum, Khartoum, Sudan; 3 Department of Pediatrics, Unaizah College of Medicine and Medical Sciences, Qassim University, Unaizah, 56219 Saudi Arabia; 4 Department of Pediatrics, College of Medicine, Qassim University, Buraydah, Saudi Arabia; 5 Department of Obstetrics and Gynecology, Unaizah College of Medicine and Medical Sciences, Qassim University, Unaizah, Saudi Arabia

**Keywords:** *Helicobacter pylori*, Adolescent, Age, Prevalence, Sudan

## Abstract

**Background:**

Only few data have been published on *Helicobacter pylori* infection in adolescents in Sub-Saharan Africa, including Sudan. The aim of the present study was to investigate the prevalence and associated factors of *H. pylori* infection in asymptomatic adolescents schoolchildren (aged 10–19 years) in Sudan.

**Methods:**

A cross-sectional study was conducted from October to November 2022. The participants’ sociodemographic and clinical characteristics were assessed using a questionnaire. The participants underwent a rapid *H*. *pylori* antibody test for the detection of *H. pylori* antibodies. Multivariate regression analyses were performed.

**Results:**

Of the 368 enrolled adolescents, 155 (42.1%) and 213 (57.9%) were boys and girls, respectively. The median (interquartile range [IQR]) age of the total sample was 15.2 years (14.0‒16.4 years). The overall prevalence of *H. pylori* infection was 8.4%. In the multivariable regression analyses, only the female adolescents (adjusted odds ratio [AOR], 3.04; 95% confidence interval [CI], 1.24‒7.44) were associated with *H. pylori* infection. Age, parental education and occupation, and body mass index were not associated with contracting *H. pylori* infection.

**Conclusion:**

*H. pylori* infection was detected in one of 10 adolescents in Northern Sudan. Female adolescents were at a higher risk of contracting *H. pylori* infection. The introduction of interventional health programs such as awareness campaigns and improving personal hygiene could lead to the reduction of the risk of *H. pylori* infection at early ages, especially in girls, and ensure that adolescents are healthy in their present and later lives.

## Introduction

The widespread global prevalence of *Helicobacter pylori* infection has conferred a heavy burden on health systems, especially in fragile health systems such as those in Africa [1, 2]. According to a recent estimation, Africa had the highest pooled prevalence of *H. pylori* infection (70.1%) compared with other regions [2]. *H. pylori* infection is still highly prevalent in children and adolescents globally [3]. Despite the descending trend in the prevalence of *H. pylori* infection in children worldwide, its overall prevalence in Africa remains very high (70%) [4]. Knowledge of the trend of the prevalence of *H. pylori* infection in children and adolescents is crucial in predicting its related diseases, including gastric cancer, in later life [4, 5]. *H. pylori* infection in children and adolescents has been associated with various extragastric pathologies such as iron deficiency anemia [6–8], growth retardation, and diabetes [8]. In addition, *H. pylori* is listed as a group 1 carcinogen in the most recent medical literature and is one of the most significant infectious drivers of cancer worldwide, accounting for 8 of 10 stomach cancer cases in adults [9]. *H. pylori* is a gram-negative bacterium with a helical shape [10].

The World Health Organization (WHO) defines adolescence as the phase of life between childhood and adulthood, from the ages of 10 to 19 years [11]. It is a unique stage of human development and an important time for laying the foundations of good health [11].

The prevalence of *H. pylori* infection in children and adolescents varies among different populations in Africa [12–16]. Moreover, several risk factors such as male sex [12, 13], age [15, 17], parental education [18] and employment [16], body mass index (BMI) [19] are associated with *H. pylori* infection in children and adolescents.

Increased awareness, appropriate screening, early identification, and individualized treatment approaches for *H. pylori* infection at early ages (children and adolescents) provide opportunities to avoid its complications [5]. These preventive approaches are needed in Africa, especially Sudan, where resources are limited. According to the WHO’s estimate, more than 20% of the population in Sudan are adolescents [20].

To attain good health, adolescents’ health must be addressed at all levels and by all involved parties. The health burden of *H. pylori* infection in adolescents requires urgent public health action. Therefore, the practical steps to address *H. pylori* infection and its associated factors in adolescents on a global scale require a thorough understanding of the local context. To achieve this, first, the prevalence of *H. pylori* infection in adolescents in the community and its associated factors must be investigated. On the basis of community-based data, appropriate health-care measures can then be applied accordingly to maintain good health. There is a paucity of studies on the prevalence of *H. pylori* infection and its associated factors in adolescents in Sudan in general [12], and no such studies have been conducted in the proposed area (Northern Sudan). Thus, the aim of the present study was to investigate the prevalence and associated factors of *H. Pylori* infection in asymptomatic adolescents in Northern Sudan.

## Methods

### Study area

This cross-sectional study was conducted in 368 asymptomatic adolescents (aged 10–19 years) who were attending public schools in the locality of Almatamah in Northern Sudan during the study period from October to November 2022. Almatamah, located in the River Nile State, Northern Sudan, is approximately 130 km from Khartoum, the capital of Sudan. Public schools for both boys and girls are available in this locality. A list of students between the ages of 10 and 19 years was obtained from the local authority, from which participants for the study were randomly selected.

### Inclusion and exclusion criteria

All adolescents between the ages of 10 and 19 years were included in the study, and those younger or older than these ages were excluded. Those who did not give their consent to participate in the study were also excluded. All the participants recruited were apparently healthy, and those who were sick, pregnant, and nursing were excluded from the study.

### Data collection

After the participants and their guardians signed an informed consent form, the selected students were approached. Data on the participants’ sociodemographic characteristics, including age in years, sex, parental educational level (< secondary or ≥ secondary), mother’s occupational status (housewife or employed), and father’s education (laborer or skilled worker), were collected using a questionnaire. Anthropometric measurements (i.e., weight and height) were performed using standard procedures.

### Weight and height measurements

The participants’ weights were measured in kilograms using standard procedures (well-calibrated scales adjusted to zero before each measurement). Weight was measured to the nearest 100 g. During the measurement, the participants stood with minimal movement, with their hands at their sides. Moreover, shoes and excess clothing were removed. Height was measured to the nearest 0.1 cm, with the participant standing straight, with their back against the wall and feet together. BMI was determined in accordance with the participant’s age, sex, weight, and height.

### Processing of blood samples

Each participant provided 3–5 ml of blood sample for *H*. *pylori* serological testing. The blood samples were centrifuged at 1,500 rpm for 15 min, and an *H*. *pylori* antibody rapid test was performed in accordance with the manufacturer’s instructions (Hangzhou Alltest Biotech Co., Ltd.; Hangzhou, China). A *H. pylori* antibody rapid test is a rapid visual immunoassay for the qualitative detection of IgM and IgG antibodies specific to *H. pylori*. It is widely used and has high sensitivity and specificity [17]. In this work, we defined our results as positive and negative for *H. pylori* infection.

### Sample size calculation

A total of 368 adolescents were enrolled in the study. We assumed that 40.0% of the adolescents had *H. pylori* infection. This assumption was based on a previous study in our neighboring country of Ethiopia [17]. The sample size of 368 adolescents was calculated to detect a difference of 5% at *α* = 0.05, with a power of 80%. We assumed that 10% of the participants would not respond or would have incomplete data.

### Statistical analysis

The data collected were entered into a computer using the IBM Statistical Package for the Social Sciences (SPSS) for Windows (version 22.0; SPSS Inc., New York, NY). The proportions were expressed as frequencies (%). The continuous data (age and BMI) were evaluated for normality using the Shapiro-Wilk test and were found to be non-normally distributed and expressed as median (interquartile range [IQR]). A univariate analysis was performed with *H. pylori* infection as the dependent variable and age, sex, parental educational level and occupation, and BMI as independent variables. Thereafter, all the variables were entered into a multivariable logistic regression model with backward elimination to adjust for covariates. The adjusted odds ratios (AORs) and 95% confidence intervals (CIs) were calculated as necessary. A two-sided *P* value of < 0.05 was considered statistically significant.

## Results

Of the 368 enrolled adolescents, 155 (42.1%) and 213 (57.9%) were boys and girls, respectively. The median (IQR) age of the total adolescents was 15.2 years (14.0‒16.4 years). The overall prevalence of *H. pylori* infection was 8.4%. Of the 368 adolescents, 231 (62.8%) and 247 (67.1%) had educated mothers and fathers (≥ secondary), respectively. Approximately 1 of 10 mothers were employed. The fathers of 147 (39.9%) of the 368 adolescents were skilled workers.

In the univariate analysis, age, parental education and occupation, and BMI were not associated with *H. pylori* infection (Table 1). In the multivariable regression analysis, only the female adolescents (AOR, 3.04; 95% CI, 1.24‒7.44) were associated with *H. pylori* infection (Table 2).

**Table 1:** Univariate analysis of the factors associated with *H. pylori* infection in adolescents in northern Sudan (n = 368), 2022

Variable		Total (n = 368)	Adolescents with *H. pylori* infection (n = 31)	Adolescents without *H. pylori* infection (n = 337)	Odds ratio (95% confidence interval)	*P* value
		Median (interquartile range)				
Age (years)		15.2 (14.0‒16.4)	15.3 (14.3‒16.4)	15.2 (14.0‒16.3)	0.92 (0.73‒1.17)	0.540
Body mass index (kg/m^2^)		18.4 (16.4‒21.4)	18.4 (16.4‒21.4)	18.5 (16.4‒21.5)	1.00 (0.90‒1.10)	0.950
		Frequency (proportion)				
Sex	Male	155 (42.1)	7 (22.6)	148 (43.9)	Reference	0.026
	Female	213 (57.9)	24 (77.4)	189 (56.1)	2.68 (1.12‒6.40)	
Mother’s Education	≥Secondary	231 (62.8)	19 (61.3)	212 (62.9)	Reference	0.859
	<Secondary	137 (37.2)	12 (38.7)	125 (37.1)	1.07 (0.50‒2.28)	
Father’s education	≥Secondary	247 (67.1)	19 (61.3)	228 (67.7)	Reference	0.471
	<Secondary	121 (32.9)	12 (38.7)	109 (32.3)	1.32 (0.61‒2.81)	
Mother’s occupation	Employed	33 (9.0)	3 (9.7)	30 (8.9)	Reference	0.885
	Housewife	335 (91.0)	28 (90.3)	307 (91.1)	0.91 (0.26‒3.17)	
Father’s occupation	Laborer	221 (60.1)	20 (64.5)	201 (59.6)	Reference	0.597
	Skilled worker	147 (39.9)	11 (35.5)	136 (40.4)	0.81 (0.37‒1.75)	

**Table 2:** Multivariate logistic regression analysis of the factors associated with *H. pylori* infection in adolescents in Northern Sudan (n = 368), 2022

Variable		Odds ratio (95% confidence interval)	*P* value
Age (years)		0.87 (0.67‒1.14)	0.325
Body mass index (kg/m^2^)		1.01 (0.90‒1.12	0.874
Sex	Male	Reference	0.014
	Female	3.04 (1.24‒7.44)	
Mother’s education	≥Secondary	Reference	0.901
	<Secondary	1.05 (0.43‒2.59)	
Father’s education	≥Secondary	Reference	0.430
	<Secondary	1.43 (0.58‒3.51)	
Mother’s occupation	Employed	Reference	0.969
	Housewife	1.02 (0.28‒3.69)	
Father’s occupation	Laborer	Reference	0.493
	Skilled worker	0.76 (0.34‒1.66)	

## Discussion

The main finding of the present study was that 8.4% of the enrolled adolescents had *H. pylori* infection. In this study, the prevalence (8.4%) of *H. pylori* infection was comparable with the results from Tanzania in children aged 6 months to 17 years, of whom 11.5% had *H. pylori* infection [16]. Moreover, in Ghana, a cross-sectional study that included 240 asymptomatic children aged 7 to 16 years reported a prevalence rate of 14.2% for *H. pylori* infection [13].

The prevalence rate (8.4%) of *H. pylori* infection in the present study is lower than that reported among 431 schoolchildren (aged 8–18 years) in Eastern Sudan, which showed that 21.8% of them had *H. pylori* infection [12].

Compared with those in other countries, the prevalence rate of *H. pylori* infection in our study was lower than that previously reported in Ethiopia (65.7%) [17] and Nigeria (32.8%) [14]. Data from a recent systematic review meta-analysis that included 198 studies with a total of 152,650 children aged ≤ 18 years showed that the overall global prevalence rate of *H. pylori* infection in children was 32.3%, which was higher in low- and middle-income countries than in high-income countries (43.2% vs. 21.7%) [3].

The low prevalence of *H. pylori* infection in our study could be explained by the fact that only asymptomatic adolescents were screened in our study. For example, a recent study by Balas et al. showed a low prevalence rate (14.2%) of *H. pylori* infection in children without gastrointestinal symptoms [4]. Moreover, the difference in the sanitation and hygiene practice in the different population could explain the difference in the prevalence of *H. pylori* infection which was reported in the different studies.

In the present study, the risk of contracting *H. pylori* infection was three times higher among the female than among the male adolescents. This is in agreement with previous studies [13, 21]. For example, in Ghana, a study by Awuku et al. that included 240 asymptomatic children revealed that a higher proportion of females than males had *H. pylori* infection (16.8% vs. 10.7%) [13]. Furthermore, Mehata et al. analyzed data from the 2016 Nepal National Micronutrient Status Survey, which included 1,023 adolescents aged 10–19 years, and revealed that 16% of adolescent girls and 14% of adolescent boys had *H. pylori* infection [21]. In Iran, a longitudinal cohort study that included 54 patients diagnosed with gastric cancer reported a significant association between female sex and higher gastric cancer stage [22]. It is not clear why females were at higher risk to have *H. pylori* infection in this study. Perhaps, there was a difference in the hygiene and sanitation practice between males and females in the region of Sudan.

Contrary to our study, a study in Eastern Sudan showed that boys were more likely to have *H. pylori* infection than girls [12]. However, other studies have shown no significant differences in the prevalence of *H. pylori* infection between boys and girls [18, 23].

Similar to our study, other studies have shown that parental educational status is not associated with *H. pylori* infection in adolescents [13]. Wangda et al., reported that the prevalence of *H. pylori* infection inversely correlated with the mother’s educational level [18]. The lack of parental education in our study could be explained by the low quality of education and the low impact of education on the workforce in Sudan.

Our study showed no association between *H. pylori* infection and father’s occupation in the enrolled adolescents, similarly to previous studies. By contrast, a study in Tanzania reported that father’s occupation was an independent predictor of the presence of *H. pylori* antibodies [16].

The present study did not show an association between *H. pylori* infection and BMI. In a recent meta-analysis, *H. pylori* infection had an adverse impact on growth outcomes in children, mainly children’s height-for-age *Z* (HAZ) scores [19]. In Eastern Sudan, where a high prevalence of *H. pylori* infection was found (21.8%), most children (84%) had BMI scores below the normal range [12].

Our study also showed no association between adolescent age and *H. pylori* infection, consistent with previous studies [12, 18]. For example, Abbas et al. revealed that *H. pylori* infection was prevalent among schoolchildren of all age groups in Eastern Sudan [12]. However, in Ethiopia [17], Nigeria [15], and Tanzania [16], older adolescent age was associated with *H. pylori* infection.

Our results should be cautiously compared with the findings of other studies for the following reasons: First, the inclusion and exclusion criteria used differed between our study and other studies. For example, asymptomatic adolescents were screened in our study, whereas adolescents with gastrointestinal symptoms were included in other studies. Second, whereas our study used the WHO cutoff age (10–19 years), other studies used different ages (early adolescence, late adolescence, or both adolescence and childhood). Third, different tests were used to detect *H. pylori* infection. Such differences in the prevalence and risk factors of *H. pylori* infection between and within countries necessitate further research.

### Limitations

This study has limitations inherent to its cross-sectional design and the direction of the association can’t be ascertained. A longitudinal study will provide more clarification regarding the association between the prevalence of *H. pylori* infection and its associated factors in later life. In Africa, *H. pylori* infection occurred in early life and carried- out in the later age. This study was conducted in Northern Sudan, thus limiting the generalization of the findings to adolescents in Sudan. One of the major limitations of the study is the lack of comprehensive risk factors (variables) to look their impact on the prevalence of *H. pylori*. We have only few socio demographic factors that are not even consider important predicators for *H. pylori* infection. The impact of WASH (Water, Sanitation, and Hygiene) interventions as potential risk factors for *H. pylori* are well-known. Moreover, this study uses rapid antibody test which can’t differentiate past and current infection.

## Conclusion

*H. pylori* infection was detected in one of 10 adolescents in Northern Sudan. The female adolescents were at a higher risk of contracting *H. pylori* infection than the male adolescents. The introduction of interventional health programs such as awareness campaigns and improving personal hygiene could lead to the reduction of the risk of *H. pylori* infection at early ages, especially in females, and ensure that adolescents are healthy in their present and later lives.

## Data Availability

The datasets used and/or analysed during the current study available from the corresponding author on reasonable request.
